# Mathematical Modeling and Inference of Epidermal Growth Factor-Induced Mitogen-Activated Protein Kinase Cell Signaling Pathways

**DOI:** 10.3390/ijms251810204

**Published:** 2024-09-23

**Authors:** Jinping Feng, Xinan Zhang, Tianhai Tian

**Affiliations:** 1 School of Mathematics and Statistics, Henan University, Kaifeng 475001, China; fjinping@henu.edu.cn; 2 School of Mathematics and Statistics, Central China Normal University, Wuhan 430079, China; xinanzhang@mail.ccnu.edu.cn; 3 School of Mathematics, Monash University, Melbourne 3800, Australia

**Keywords:** mitogen-activated protein kinase, epidermal growth factor, mathematical model, cell signaling pathway, single cell, inference

## Abstract

The mitogen-activated protein kinase (MAPK) pathway is an important intracellular signaling cascade that plays a key role in various cellular processes. Understanding the regulatory mechanisms of this pathway is essential for developing effective interventions and targeted therapies for related diseases. Recent advances in single-cell proteomic technologies have provided unprecedented opportunities to investigate the heterogeneity and noise within complex, multi-signaling networks across diverse cells and cell types. Mathematical modeling has become a powerful interdisciplinary tool that bridges mathematics and experimental biology, providing valuable insights into these intricate cellular processes. In addition, statistical methods have been developed to infer pathway topologies and estimate unknown parameters within dynamic models. This review presents a comprehensive analysis of how mathematical modeling of the MAPK pathway deepens our understanding of its regulatory mechanisms, enhances the prediction of system behavior, and informs experimental research, with a particular focus on recent advances in modeling and inference using single-cell proteomic data.

## 1. Introduction

The mitogen-activated protein kinase (MAPK) pathway is a crucial intracellular signaling pathway that plays a pivotal role in several cellular processes, including cell differentiation, proliferation, apoptosis, and survival [1,2,3]. Highly conserved across eukaryotes, this pathway is important for regulating various developmental and physiological processes [4]. Dysregulation of the MAPK pathway is associated with various diseases, including cancer, neurodegenerative disorders, and inflammatory conditions [5,6]. Consequently, understanding its functions and regulatory mechanisms is critical for developing targeted interventions and therapies [7,8,9,10].

Typically, the MAPK pathway is activated in response to extracellular signals, such as growth factors, cytokines, and stress stimuli. Activation begins when extracellular ligands bind to cell surface receptors like receptor tyrosine kinases, causing a conformational change in the receptors [11,12]. The activated receptors initiate downstream protein kinases, which trigger the small GTPase protein Ras. The Ras–Raf–MEK–ERK module is a well-studied MAPK pathway. Activated Ras activates Raf protein by recruiting it from the cytosol to the plasma membrane [13,14]. Once activated, Raf kinase activates MAP/ERK kinase (MEK), which then activates extracellular signal-regulated kinase (ERK) by phosphorylation. The activated ERK translocates to the nucleus, where it phosphorylates a number of substrates, leading to changes in gene expression that finally influence cellular processes [15,16]. Research has demonstrated how positive feedback in the pathway regulates switch-like output, negative feedback causes gradient response, and coherent feedforward loops generate and determine cellular memory [17].

The MAPK pathway also engages in crosstalk with other signaling pathways to regulate diverse cellular functions [18]. The other two MAPK modules, p38 and c-Jun N-terminal kinase (JNK), interact with the ERK pathway. JNK and p38 kinases are generally linked to stress responses and apoptosis [19]. Additionally, phosphoinositide 3-kinase (PI3K) and the protein kinase B (AKT) pathway, as well as the mammalian target of the rapamycin (mTOR) pathway, interact with the MAPK pathway [20,21]. Other pathways, including the Wnt signaling pathway and the Notch signaling pathway, also crosstalk with the MAPK pathway at various levels [22,23]. Understanding these interactions is essential for decoding the complexity of cellular responses.

Mathematical modeling of the MAPK pathway develops networks and equations to represent biochemical interactions between the major components in the pathway [24,25,26]. Two major modeling methods for describing signaling networks are dynamical modeling and graph theory. In the graph theory category, effective methods have been developed using statistical techniques, information theory, and machine learning algorithms [27,28,29,30]. This review focuses on dynamic models that provide insights into cellular responses, signal transduction dynamics, and regulatory relationships in the MAPK pathway [31,32]. Computer simulations enable the exploration of dynamic behavior under various perturbations and experimental conditions [33]. In recent years, machine learning algorithms have been employed to tackle the challenges associated with proteomic datasets and to describe signal information transmission that is functionally pleiotropic [34]. Mathematical modeling has significantly advanced our understanding of MAPK’s regulatory mechanisms, improved the prediction of system dynamics, and offered valuable guidance for experimental design [35].

Recent advances in proteomic technologies, particularly single-cell proteomics, allow us to measure the status of dozens of proteins in single cells concurrently [36,37]. These developments provide unprecedented opportunities to study protein phosphorylation dynamics and investigate the heterogeneity of multi-signaling networks inside cells [38,39,40]. To explore cellular variability, signaling plasticity, and collective responses, several single-cell analytical techniques have been developed, including bioinformatics software, mathematical models, and statistical inference methods [41,42,43,44,45,46,47]. In addition to cellular heterogeneity, biological noise contributes to variability in gene expression, protein levels, and cellular behavior. This noise can be classified into intrinsic and extrinsic types [48,49]. Intrinsic noise refers to the stochasticity of biochemical interactions during molecular processes within the network, whereas extrinsic noise arises from other cellular processes or environmental fluctuations [50,51]. Noise in signaling networks may also arise from the inherent promiscuity of protein–protein interactions (PPIs), shaping cellular signal transduction [52,53]. Both deterministic and stochastic models provide a powerful framework for integrating these factors and predicting the outputs of system interventions and perturbations in both healthy and diseased cells [54,55,56]. Despite these advancements, analyzing large-scale single-cell proteomic data and constructing mechanistic models remains significantly challenging [57].

This review provides a comprehensive overview of the mathematical modeling of the MAPK pathway, with an emphasis on dynamic models for signaling in healthy cells. Section 2 begins by discussing mathematical models for the Ras–Raf–MEK–ERK module. Section 3 reviews models of the receptor-activated MAPK pathway. In Section 4, we explore models addressing crosstalk between the MAPK pathway and other signaling pathways. Section 5 summarizes data-driven modeling approaches, followed by an overview of recent advances in single-cell data and stochastic modeling for cellular heterogeneity in Section 6. Section 7 discusses inference methods for estimating unknown parameters. Finally, we address the challenges of modeling the MAPK pathway using single-cell data.

## 2. Modeling of MAP Kinase Modules

**Figure 1:**
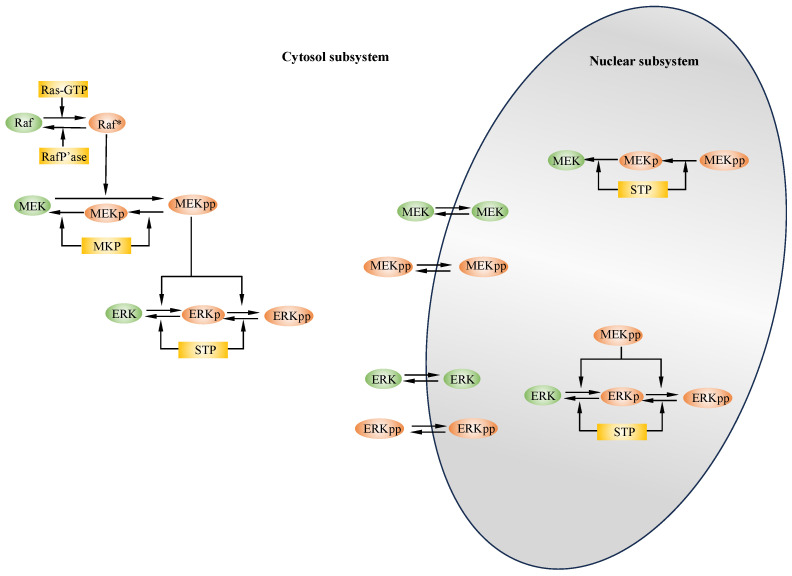
Schematic overview of the Ras–Raf–MEK–ERK module: In the cytosolic subsystem, the Ras–Raf–MEK–ERK pathway begins when the input signal Ras–GTP activates Raf, which subsequently activates MEK through a single-step processive module. MEK then activates ERK kinase in a two-step distributive manner. Both active and inactive forms of MEK and ERK are capable of freely diffusing between the cytosol and the nucleus. In the nuclear subsystem, activated MEK can further activate ERK. Specific phosphatases, such as Raf phosphatase, MKP, and STP, deactivate the active forms of Raf*, MEKpp, and ERKpp at various subcellular locations [71].

Huang and Ferrell pioneered the first mathematical model using ordinary differential equations (ODEs) to predict the highly ultrasensitive response of the MAP kinase cascade [58]. This cascade comprises MAP kinase kinase kinase (M3K), MAP kinase kinase (M2K), and MAP kinase (M1K), corresponding to Raf, MEK, and ERK, respectively, as shown in Figure 1. Subsequent studies have investigated how positive and negative feedback loops generate signal outputs with properties such as ultrasensitivity, multistability, and oscillation in both mammalian cells [59,60,61] and yeast [62,63]. Additionally, mathematical analyses have examined the roles of scaffolding proteins, phosphatases, and feedback interactions in modulating the duration and amplitude of signal outputs [64,65]. The structure of tiered kinase modules and the nature of processive and distributive phosphorylation reactions have been shown to be crucial for producing robust graded or switch-like signal outputs [66]. Both mathematical modeling and experimental validation have demonstrated that the ERK pathway acts as a negative feedback amplifier, converting intrinsic switch-like activation kinetics into graded linear responses [67]. However, experimental evidence has shown that ERK kinase is phosphorylated processively in HeLa cells due to molecular crowding [68]. Furthermore, Bayesian inference methods have been developed to estimate unknown model parameters, facilitating the realization of diverse signal outputs or multisite phosphorylation [69,70].

Signaling pathways typically begin with transmembrane receptors, making membrane components essential for MAP kinase signal transduction [72]. Experimental studies have indicated that the MAP kinase module at the plasma membrane can produce maximal outputs in response to subtle analog input signals, which differ from those generated in the cytosol [73]. Additionally, Ras nanoclusters, consisting of five or more Ras proteins, act as sensitive switches, converting graded ligand inputs into fixed outputs of activated ERK [74]. A stochastic model has been developed to explore the role of K-Ras nanoclusters formed on the plasma membrane in the signal transduction of the MAPK module [75]. This model was used to simulate the coupling between membrane proteins in different leaflets to examine how signals are relayed from the outer to the inner leaflet of a cellular membrane [76]. Another study investigated how ligand-induced higher-order epidermal growth factor receptor (EGFR) oligomerization or clustering, distinct from EGFR dimers, influenced signaling outputs [77].

The movement of kinases between the cytosol and nucleus can alter signal output patterns. Both sustained and pulsed growth factor stimulations have revealed novel features of MAPK network topology and heterogeneous ERK dynamics [78]. Sustained oscillations of ERK kinases, with a periodicity of approximately 15 min, have been observed between the nucleus and cytoplasm, persisting for over 45 cycles [79]. The nuclear translocation of kinase activities and the temporal dynamics of the three MAPK modules have been observed in living cells [80]. Building on Huang and Ferrell’s classic model [58], oscillatory signal outputs have been achieved by varying model parameters or signal input [69,81]. A kinetic model, consisting of the essential members of the Ras–ERK cascade, has been developed to reproduce the key features of ERK activation and nuclear translocation observed experimentally [82]. Furthermore, both experimental and mathematical studies have demonstrated that nuclear translocation and auto-regulation can convert graded ERK signal outputs into switch-like responses [83]. A recent study examined how receptor-driven ERK pulses reconfigure MAPK signaling, thereby enhancing the adaptability and resilience of MAPK signaling in the context of targeted cancer therapies [84].

## 3. EGFR-Induced MAPK Pathway

**Figure 2:**
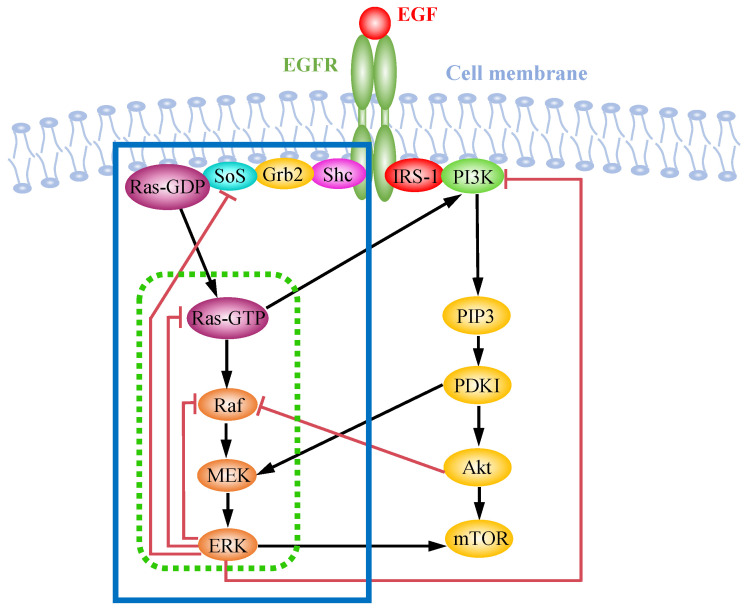
Schematic overview of the MAP kinase pathway and the PI3K/AKT pathway activated by EGF receptors. The box with the green dashed line includes the Ras–Raf–MEK–ERK module, as shown in Figure 1, while the box with the blue solid line encompasses the EGF-induced MAPK pathway.

The EGFR-induced MAP kinase pathway is a critical signaling cascade that transmits extracellular signals into the cell, influencing key processes such as proliferation, growth, survival, and differentiation [85]. This pathway is triggered when EGF binds to the extracellular domain of EGFR, promoting receptor dimerization and activation through autophosphorylation (illustrated on the left side of Figure 2). Once phosphorylated, EGFR forms signaling complexes by interacting with downstream proteins such as Shc, Grb2, and SOS. The activation of SOS kinase subsequently activates Ras, which, in turn, activates the MAPK module, as shown in Figure 1 [86]. Experimental studies have demonstrated that a series of feedback regulatory loops modulate the MAPK pathway, leading to diverse signal outputs [87].

One of the earliest mathematical models of this pathway was developed to explore how signal outputs vary in response to different types of signal inputs [88]. A subsequent study modeled the interactions between the MAPK pathway and the protein kinase C (PKC) signaling pathway [89]. To better understand signal modulation in response to transient stimuli, a mathematical model was developed for the quantitative analysis of cellular signal transduction in the EGF-activated pathway [90]. A comprehensive model of EGFR signaling was also developed, encompassing both the Shc-dependent and Shc-independent pathways, as well as receptor internalization [91]. Different EGFR ligands have been shown to result in varying receptor dimerization strengths and signaling dynamics [92], including FGF2 binding to FGFRs and heparan sulfate proteoglycan (HSPG) co-receptors [93]. High-throughput imaging of individual cells has been employed to study how transient extracellular growth factor signals are processed by the EGF–MAPK pathway [94]. Numerous mathematical models have been developed to build a theoretical framework for analyzing the specificity and fidelity of signaling networks [95,96]. To accurately quantify kinase dynamics, RNA-seq and proteomic datasets have been used to determine the absolute abundance of 16 core proteins and 10 feedback regulators in the MAPK pathway across normal and breast cancer cell lines, as well as fibroblasts [97]. Another study measured 43 phosphopeptides from the EGFR–MAPK pathway [98].

The initial signaling mechanism of the EGFR pathway involves ligand-induced, receptor-mediated dimerization and activation of EGFR [99,100,101]. Mathematical models have been employed to investigate the impact of the dimerization mechanism on VEGF binding to cell surfaces and the formation of active receptor complexes [102]. A stochastic spatial model was developed to simulate EGFR diffusion and dimerization [103], and a quantitative experimental framework and kinetic model were developed to identify mechanisms of receptor tyrosine kinase (RTK) reprogramming [104]. The next key mechanism involves EGFR phosphorylation-dependent endocytosis [105,106]. HER2, a member of the EGFR tyrosine kinase family, also plays a role in mediating EGFR trafficking [107]. Further modeling studies have explored the kinase functions within the pathway, including the role of Shp2 phosphorylation and the crosstalk between the RhoA and EGFR–ERK pathways [108,109].

Understanding the influence of both positive and negative regulation on signal output is crucial in experimental and computational studies [110]. Deterministic and stochastic models have been used to study ERK-mediated negative feedback regulation of SOS [111,112]. Mathematical models have also examined how interlinked positive and negative feedback loops influence the amplitude, duration, and frequency of ERK pulses [113,114]. Another study investigated how these feedback loops contribute to bistability and oscillatory signal output [115]. Both experimental and modeling studies have demonstrated that the strong negative feedback loop from ERK to Raf is essential for maintaining robust signal output under perturbations [116].

## 4. Crosstalk within MAPK and AKT Pathways

The EGF-induced AKT pathway (illustrated on the right side of Figure 2) is initiated when EGF binds to EGFR, recruiting phosphoinositide 3-kinase (PI3K) via adaptor proteins such as IRS-1 and Gab. PI3K activation results in the conversion of PIP2 to PIP3, leading to the activation of PDK1, which, in turn, activates AKT through phosphorylation [117]. A comprehensive overview of the crosstalk between the MAPK and AKT pathways can be found in [118]. The mTOR pathway acts as a downstream target of the AKT signaling, promoting proliferation, cell growth, and protein synthesis [119,120].

Hatakeyama et al. developed an early mathematical model to explore the crosstalk between the MAPK and AKT pathways [121], which is based on experimental evidence showing negative regulation from AKT to Raf kinase. A sensitivity analysis of this model identified key parameters influencing pathway dynamics [122,123]. A Bayesian approach was employed to infer model parameters, testing the hypothesis of positive regulation from PDK1 to MEK, alongside the established negative feedback from AKT to Raf and positive feedback from PI3K to Raf [124]. Subsequent in silico analysis suggested that the Raf and AKT pathways function independently [125].

A comprehensive mechanistic model was developed to predict cellular responses to EGF under various perturbations, indicating that the temporal dynamics of Gab1 are significantly controlled by positive and negative feedback loops [126]. A detailed ODE system, consisting of 478 differential equations, was constructed to quantify signal flow in the MAPK and AKT pathways and their crosstalk [127]. Another mathematical model was designed to identify key interaction parameters and feedback loops between these two critical signaling pathways, determining both normal and disease phenotypes [128]. Feedforward and feedback regulation, as well as pathway crosstalk, were identified as key mechanisms generating experimentally observed oscillations in MAPK and PI3K signaling [129]. A phosphoproteomics-based study measured activities and perturbations of 38 key kinases in the EGFR signaling pathway [130].

Crosstalk between signaling pathways has also been examined in networks induced by other growth factors, including the ErbB signaling activated by EGF and heregulin (HRG) [131], as well as the activation of the MAPK and AKT pathways by EGF and insulin growth factor (IGF) [132]. Crosstalk has been observed between the pathways by the activation of platelet-derived growth factor (PDGF) [133], as well as between the ERK, AKT, and Wnt/β-catenin signaling pathways activated by EGF and the Wnt-protein ligand [134]. Additionally, an extensive logical model has been developed to illustrate the crosstalk between three MAPK modules (ERK, JNK, p38), the AKT pathway, and the PKC pathway, all of which are activated by various ligands [135]. Recent studies have provided enriched data and new insights into regulatory mechanisms, such as those governing the PTEN/AKT-β-catenin pathway and its crosstalk with the MAPK pathway [136,137]. Further, mathematical models are needed to explore the crosstalk between the MAPK pathway and other pathways, such as Wnt, Notch, and TNF-alpha [138,139].

## 5. Data-Driven Modeling of Signaling Pathways

Mathematical models using ODEs have been developed to study the MAPK pathway with proteomic data. An early study inferred an ODE model of the MAPK module, including a nuclear subsystem, using proteomic datasets and experimental data on Ras activities as pathway inputs [140,141]. The integration of Western blotting analysis data improved the model’s prediction accuracy [82]. The BioNetGen language was used to develop a model to rank protein–protein interactions at the EGFR level on the plasma membrane, based on proteomic data from 11 different cell lines also from Mann’s group [142,143,144]. Another ODE model system, developed using proteomic, transcriptomic, and imaging data from melanoma cells, identified two parallel MAPK reaction channels that are differentially sensitive to RAF and MEK inhibitors [145]. Additionally, a multi-omics integration model combined RNA-sequencing data [146] and proteomic data [97] to predict the coordinated dynamics of the ERK and AKT pathways in response to synergistic mitogen or drug combinations [147]. Stochastic simulations suggested that ERK dynamics, but not AKT, may drive variability in cell proliferation [147]. Furthermore, a comprehensive mechanistic signaling model called SPARCED was developed to explore the crosstalk between EGF-induced pathways and the IFNγ pathway [148]. Using extensive experimental data (i.e., 200 sets) from both the literature and in-house experiments, a comprehensive model was developed that included 67 molecular species from various signaling pathways, such as the three MAPK modules, as well as the AKT, NF-κB, and JAK/STAT pathways [149]. This model serves as an initial network-centric, comprehensive “virtual macrophage” simulation platform.

**Figure 3:**
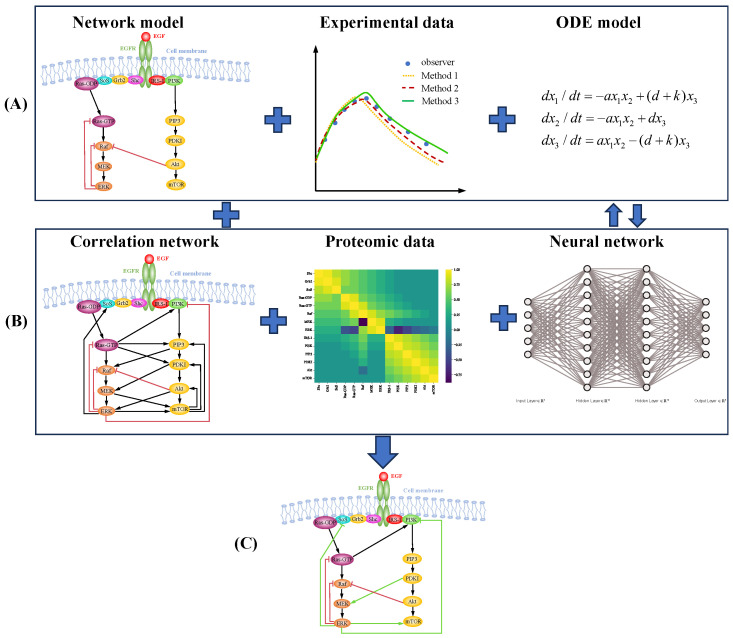
Mechanistic and data dual-driven approaches for modeling cell signaling pathways. (**A**) Mechanistic modeling approaches rely on experimentally discovered regulatory mechanisms, kinase activity data, and dynamic models to simulate cell signaling pathways. (**B**) Data-driven modeling approaches utilize static correlation network models, omics datasets, and statistical methods or machine learning algorithms to analyze signaling pathways. Two main combination techniques are employed for dual-driven approaches. The parallel structure approach uses weighting techniques to merge results from different models into a single output, whereas the serial structure approach uses the prediction of one model as the input for another model. (**C**) Inferred network model: The final network model is constructed by integrating predictions from the dual-driven approaches.

A hybrid modeling approach, also known as multi-scale or dual-driven modeling, integrates mechanistic models to quantify partially understood processes with data-driven models to explore unknown processes [150,151,152,153] (see Figure 3). Two main methods for integrating results from different models are illustrated in Figure 3 [154]. Statistical regression methods and artificial neural network algorithms are widely used in dual-driven modeling [155]. For the MAPK pathway, the MEMMAL framework combines mechanistic modeling and machine learning to create computational models from multi-omics datasets [156]. Another dual-driven modeling approach combined mechanistic models with partial least-squares regression (PLSR) to predict cell responses to different ligands in the EGFR pathway [157].

In addition to the mechanistic ODE models discussed above, other dynamic modeling methods have been used to describe regulatory mechanisms in cell signaling pathways. These include the following:

- Non-parametric approaches, which include signaling Petri net-based simulations [158].
- Discrete dynamic modeling, which does not require kinetic parameters [159].
- Dynamic logical network models, which use Boolean variables for MAPK modules and the AKT and P53 pathways [160,161].
- Fuzzy logic approaches, which predict signaling crosstalk evolution in response to perturbation and over time [162,163].
- The BowTieBuilder pipeline, which is used to infer signal transduction pathways [164].
- Information theory-based methods, which analyze signaling pathways [165].
- Sensitivity, robustness, and fragility analyses, which assess signaling pathway properties under perturbation [166,167,168].
- Extended Boolean network models, which incorporate stochastic processes [169].
- cSTAR (Cell-State Transition Assessment and Regulation), which transforms omics data into input for mechanistic models [170].
- Non-Markovian signaling processes, which account for signaling intermediates with random time delays [171].

It is worth noting that developing multi-scale models for cell signaling pathways by combining these diverse dynamic models is essential for capturing the complexity of cellular signaling.

## 6. Stochastic Models for Cell Signaling Pathways

**Figure 4:**
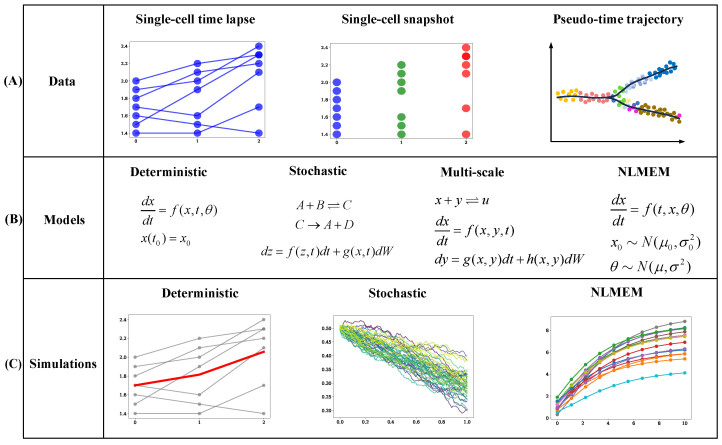
Modeling and simulation of cell signaling pathways using single-cell data. (**A**) Data types: Single-cell data include time-lapse and snapshot proteomic data, with pseudo-time trajectories generated from snapshot data using bioinformatics methods. (**B**) Model types: Stochastic models may involve chemical reaction systems (CRS) or stochastic differential equations (SDEs). Multi-scale models combine multiple model types, such as ODEs, CRS, and SDEs. (**C**) Simulation types: Stochastic simulations can be generated from either stochastic models or multi-scale models. (Red-line in deterministic: the average simulation of all simulations; lines in stochastic and NLMEM with different color: different simulations of the stochastic model and NLMEM model, respectively).

Stochastic modeling approaches, such as chemical reaction systems and stochastic differential equations (SDEs), are critical for studying the role of noise in regulatory networks [172,173,174] (see Figure 4). For instance, a stochastic model incorporating 150 chemical reactions for the EGF signaling pathway and nuclear pore complex showed how cellular noise modulates EGF signaling activity [175]. Similar results were found in an SDE model of the MAPK module, which was based on measured dynamics of phosphorylated MEK and ERK across cell populations [176]. Another SDE model revealed that 80% of ERK activity pulses were noise-driven, with the remaining 20% resulting from cell-to-cell propagation [177]. Additionally, a stochastic model with 34 chemical reactions was employed to simulate the modulation of the MAPK pathway influenced by methyltransferase 5 (PRMT5) [178]. Random models have also been used to describe kinase dynamics and cell-to-cell variability by introducing stochastic initial conditions into ODE models [179]. For downstream kinase activities, a spatial stochastic model examined the opposing roles of progesterone receptor (PR) phosphorylation mediated by ERK kinase [180]. An SDE model was also utilized to describe the temporal stochastic bursting of the TGF-β/SMAD signaling pathway in single cells [181]. Stochastic fluctuations were introduced in the reactions at the receptor level, as receptor dynamics are rate-limiting in TGF-β signaling [182].

Multi-scale models have been developed to study biological systems at various levels, from pathways and cells to tissues and whole organisms [183,184]. For example, a multi-scale model has been used to account for emergent heterogeneity resulting from intercellular signaling influences on individual cells within a population [185]. Molecule and kinase diffusion are important in stochastic modeling. A stochastic spatial model simulated receptor diffusion and EGFR dimerization [103]. Another stochastic simulator explored EGFR signal initiation by recruiting signaling molecules like Grb2 and Shc to the phosphorylated EGFR tail, influenced by receptor density, reaction kinetics, and membrane spatial organization [186]. Furthermore, a stochastic model was developed to study Ras nanocluster formation, with simulation results consistent with experimental findings regarding the role of the scaffold protein galectin-3 in nanocluster formation [75,187].

The nonlinear mixed-effect model (NLMEM) is a statistical approach for analyzing complex data with both within-subject and between-subject variability [188,189,190]. The NLMEM is particularly effective for describing deterministic and random effects within a cell, as well as variations between cells, using probability-based parameters [191,192,193]. The distribution-independent single-cell ODE modeling (DISCO) approach, based on the NLMEM framework, uses a deterministic model with distributed parameters to infer continuous single-cell signaling dynamics from multiplexed snapshot data [194]. A similar semi-deterministic modeling approach assumes that the parameters of the deterministic MAPK pathway model follow log-normal distributions [78]. ODE-constrained mixture models have been proposed to study NGF-induced Erk1/2 phosphorylation [195]. These models describe the dynamics of each subpopulation using an ODE model but with different parameters for each subpopulation. The NLMEM has also been used to integrate multiple single-cell perturbation experiments, such as studies on translation kinetics after transfection [196].

Several modeling frameworks have been proposed to study the dynamic properties of cell signaling pathways using single-cell data. The FitMultiCel pipeline l is a computationally efficient framework that can handle the entire workflow of modeling, simulating, and parameterizing multi-scale models of multi-cellular processes [197], based on tools like Morpheus [198] and pyABC [199]. Another open-source pipeline was designed for scalable, single-cell mechanistic modeling from simple annotated input files, providing a foundation for mechanistic data integration [200]. These pipelines exemplify simulation-based inference (SBI), which seeks to identify parameter sets compatible with prior knowledge and empirical observations [201,202]. A recent study compared the efficiency and accuracy of various SBI approaches, including machine learning algorithms and optimization workflows [203].

Machine learning algorithms have also been employed as data-driven approaches to visualize single-cell trajectories, identify prototypic trajectories, and extract distinctive motifs in ERK and AKT signaling [204]. Neural networks, such as those used in Density-PINNs (density physics-informed neural networks), have been applied to infer signal transduction time distributions from experimental data [205]. PINNs are valuable tools that use neural networks to solve differential equations [206]. Another data-driven approach, a multi-modal heterogeneous ensemble integration framework [207], was trained on growth factor-induced ERK and AKT activity time courses in single cells [208].

In addition to models, numerous experimental studies have collected population and single-cell data under various conditions, including BRAF inhibitor treatment in single melanoma cells [209], MEK and ERK kinase activities in PC12 cells [176], MAP kinase activities induced by EGF/NGF [78], single-cell ERK dynamics under various inhibitors and perturbations [210], NGF/TrkA-initiated ERK1/2 signaling kinase activities [211], single-cell ERK and AKT activity time course data for controlling the cell cycle [208], single-cell MAPK kinetics under computer-controlled temporal stimulations [212], crosstalk between the JNK and p38 signaling pathways [213], correlated ERK and AKT activities induced by G-protein-coupled receptors [214], ERK and AKT signaling in response to various growth factors [204], kinase activities in the three MAPK modules, the NF-κB and p38 pathways [80], and the dynamics of EGFR–Ras–ERK signaling that enhance heterogeneity in target gene expression [215]. These datasets offer rich information for mathematical modeling studies of cell signaling pathways.

The Systems Biology Markup Language (SBML), an XML-based format for computational models of biochemical networks, has become a widely accepted standard for representing and exchanging models [216,217]. Several platforms have been developed to use SBML for modeling complex molecular systems, including the MATLAB Toolbox [218,219], SBML-R interface [220], and various Python packages [221]. SBML has also been used to model and simulate the dynamics of the MAPK pathway [222,223]. Many modeling studies have published computer programs for simulating the dynamics of the MAPK pathway. Table 1 provides a list of modeling platforms and programming codes that are available for download.

## 7. Parameter Inference

The parameters of dynamic models for cell signaling pathways are rarely known a priori and must be estimated by calibrating the model using experimental data. Two major inference methods used for this task are optimization methods and Bayesian approaches [237,238]. Parameter inference is essential not only for model selection using statistical criteria but also for reverse-engineering regulatory networks with omics datasets [239,240,241]. This section focuses on the application of these inference methods to models of cell signaling pathways.

Optimization methods have been widely used to estimate unknown parameters in mathematical models of the MAPK pathway. These include the genetic algorithm implemented on a parallel platform [242], the extended Kalman filter for state and parameter estimation in the JAK–STAT and Ras/Raf/MEK/ERK pathways [243], and a scalable and parallel optimization method that is over $10^{4}$ times faster than state-of-the-art methods [244]. A modular Python framework (fides) enables systematic comparison of different approaches to ODE model calibration with various Hessian approximation schemes [245]. Additionally, a deep learning algorithm using mini-batch optimization has been applied [246].

Bayesian methods represent another major category of inference techniques for estimating unknown model parameters. Markov Chain Monte Carlo (MCMC) methods, such as the Metropolis–Hastings algorithm, have been used to estimate parameters in models of NGF- and EGF-induced ERK/AKT pathways [247] and to determine the multisite phosphorylation rate constants of ERK kinase by MEK kinase [70]. Additionally, a Bayesian inference-based modeling (BIBm) approach was developed to infer the topologies of EGF-induced ERK pathways [248]. In recent years, approximate Bayesian computation (ABC) has become widely used in systems biology modeling, as these methods do not require a likelihood function. The sequential Monte Carlo (SMC) method has been applied to develop the ERK MAP kinase model using proteomics data and Bayesian model selection criteria [249,250]. We proposed incorporating simulation errors into the criterion for sample selection in ABC-SMC [251] and designed a population-based algorithm to calibrate the Raf–MEK–ERK module [252]. Other methods, such as causal inference and information theory-based inference, have been developed to infer the network structure and the heterogeneity of signaling networks using single-cell data [253,254].

For estimating parameter distributions in NLMEMs, maximum likelihood estimation is commonly used. However, this method typically assumes that random-effect distributions are normal and independent [255,256]. Bayesian estimation provides a more flexible approach, accommodating more complex assumptions about parameter distributions in NLMEMs. A computational sampling method called “Contour Monte Carlo” (CMC) has been proposed for estimating model parameters in TNF signaling from snapshot distributions [257]. To address the computational challenges of traditional NLMEM inference methods, a subsequent approach known as filter inference was introduced, enabling efficient inferences from snapshot measurements [258]. Alternative probability distributions beyond the normal distribution, have also been explored for robust calibration of hierarchical population models [259]. Additionally, a scalable and flexible framework has been proposed for Bayesian inference in state-space mixed-effects single-cell models with stochastic dynamics [260]. A likelihood-based framework has also been developed for inference and identifiability analysis of differential equation models that account for biological heterogeneity through probabilistic parameters [261].

## 8. Discussion and Conclusions

The increasing availability of single-cell proteomic data presents both significant opportunities and substantial challenges for the mathematical modeling and analysis of heterogeneity in cell signaling pathways. These challenges primarily stem from the inherent complexity of these pathways, which involve numerous kinases, intricate regulatory mechanisms, and crosstalk between different pathways. The multi-scale nature of signaling pathways, which operate across various cellular locations, makes it difficult to capture all relevant processes within a single model. Moreover, often incomplete or sparse experimental data on signaling pathways further complicate accurate model parameterization. As signaling network models become more complex and comprehensive, advanced modeling techniques are needed to simplify these models while preserving their predictive capabilities. However, maintaining a balance between model simplicity and biological realism remains crucial. We emphasize that biological realism is crucial for the accuracy and success of mathematical modeling. Experimental studies have demonstrated that kinases exhibit different activity patterns in different cellular locations. Multi-scale and spatial modeling are essential to understanding signaling pathways that operate in various cellular locations.

The stochastic nature of chemical events, noisy single-cell data due to technical limitations, and the inherent heterogeneity of single-cell data makes it challenging to design a unified modeling framework that accounts for all these variations [262]. Unlike gene expression, which often involves species with very small copy numbers, the number of kinases in cell signaling pathways is typically large. Further research is needed to identify the origins of noise at various levels. While stochastic differential equation mixed-effects models show promise for studying intrinsic and extrinsic noise and heterogeneity simultaneously [260], applying such complex models to large-scale cell signaling pathways remains technically challenging. Assumptions regarding parameter distributions and correlations between parameters are also critical, particularly when dealing with non-Gaussian and/or non-Markov stochastic processes in signaling pathways.

Parameter inference is particularly challenging with high-dimensional single-cell data. The vast parameter search space complicates the identification of a unique parameter set, as different candidate sets can generate similar simulations that match experimental data [262]. This challenge is further amplified in stochastic models due to the limited amount of observational data, often consisting of a single set of observations, leading to greater variation in candidate estimates. Mathematical models of cell signaling pathways often include large numbers of differential equations and unknown parameters, requiring significant computational power and risking inaccuracies due to the complex search space. Existing approaches may still struggle to infer large-scale stochastic models of cell signaling pathways due to substantial computational demands. Practical solutions like early rejection algorithms can reduce computational time [27,263], while machine learning algorithms, such as PINNs [264], offer promising directions for developing effective inference methods.

Finally, our focus in this work is on the dynamic mathematical modeling of the MAP kinase pathways. We do not cover other significant topics, such as bioinformatics approaches for inferring static protein–protein interaction networks, the mathematical modeling of cell signaling networks in tumor cells with genetic mutations, or inhibitors and drug resistance. Due to space constraints and gaps in our knowledge, there are important references that we were unable to include. While we acknowledge the value of these excluded studies, any oversights or omissions are our responsibility.

## Figures and Tables

**Table 1:** Computer programs for simulating the dynamics of the MAPK pathway. All websites were accesses on 16 September 2024.

Name	Web Link	Language	Ref/Comment
MAPKcascades	https://github.com/SJHamis/MAPKcascades	MATLAB	[224]
MARM1	https://github.com/labsyspharm/marm1-supplement	Python	[84]
MRA-SMC-ABC	https://github.com/SBIUCD/MRA_SMC_ABC1	MATLAB	[225]
Modeling	https://github.com/Jia-V/modeling	MATLAB	[226]
PCC-Mutation	https://github.com/drplaugher/PCC_Mutations	MATLAB& Python	[227]
Tslearn	https://github.com/tslearn-team/tslearn	Python	[228]
Biomass	https://github.com/okadalabipr/biomass	Python	[229]
pulsatile-information	https://github.com/pawelnalecz/pulsatile-information	Python	[230]
Adaptive MPC	https://github.com/Ben-Smart/Adaptive_MPC_on_NSCLC	MATLAB	[231]
MaBoss	https://github.com/sysbio-curie/MaBoSS_test	Python	[232]
TRACT	https://github.com/developerpiru/TRACS	Python	[233]
HyMetaGrowthXTreat	https://github.com/NMDimitriou/HyMetaGrowthXTreat	MATLAB	[234]
MixedIC50	https://github.com/NKI-CCB/MixedIC50	R	[235]
MaSoFin	https://github.com/guijoe/MaSoFin	C	[236]

## Data Availability

Not applicable.

## Abbreviations

The following abbreviations are used in this manuscript:

MAPK	mitogen-activated protein kinase
JNK	c-Jun N-terminal kinase
PI3K	phosphoinositide 3-kinase
mTOR	mammalian target of rapamycin
EGFR	epidermal growth factor receptor
MEK	MAP/ERK kinase
ERK	extracellular signal-regulated kinase
AKT	protein kinase B
ODE	ordinary differential equation
SDE	stochastic differential equation
NLMEM	nonlinear mixed-effect model
ABC	approximate Bayesian computation
